# Enhancement of Cell Behavior by the Polysaccharide Extract of *Arthrospira* and Potential Biomedical Applications

**DOI:** 10.3390/molecules28020732

**Published:** 2023-01-11

**Authors:** Junpeng Xu, Shan-hui Hsu

**Affiliations:** 1 Institute of Polymer Science and Engineering, National Taiwan University, No. 1, Sec. 4 Roosevelt Road, Taipei 10617, Taiwan; 2 Institute of Cellular and System Medicine, National Health Research Institutes, No. 35, Keyan Road, Miaoli 35053, Taiwan

**Keywords:** sulfated polysaccharide, *Arthrospira*, neural stem cell, surface modification, drug carrier

## Abstract

*Arthrospira* is one of the most studied cyanobacteria and has been reported with practical applications. Among the substances derived from *Arthrospira*, polysaccharides have received relatively less attention than phycocyanins, though they have more abundant structural variations and specific properties. Herein, a new *Arthrospira*-derived sulfated polysaccharide was explored for its potential bioactive functions. The ability of this sulfated polysaccharide to promote the behavior of neural stem cells (NSCs) in three-dimensional hydrogel was examined for the first time. NSCs encapsulated in the sulfated polysaccharide-containing hydrogel showed better proliferation than the control hydrogel as well as a unique cell clustering behavior, i.e., formation of multicellular spherical clusters (40–60 μm). The sulfated polysaccharide, in an appropriate range of concentration (5 mg/mL), also maintained the stemness of NSCs in hydrogel and facilitated their differentiation. In addition, the potentials of the new sulfated polysaccharide as a coating material and as a component for drug carrier were verified. The sulfated polysaccharide-modified substrate exhibited superhydrophilicity (contact angle ~9°) and promoted cell adhesion to the substrate. Composite nanoparticles composed of the sulfated polysaccharide and other differently charged polysaccharides were produced with an average diameter of ~240 nm and estimated drug loading of ~18%. The new *Arthrospira*-derived sulfated polysaccharide is a promising candidate for cell culture, surface-modification, and drug-delivery applications in the biomedical field.

## 1. Introduction

Natural polysaccharides are usually complex polymers consisting of a group of monosaccharide units linked by glycosidic bonds isolated from renewable resources [1]. Natural polysaccharides are often linear or branched structures with molecular weights greater than 100 kDa, which maintain unique biological activity [2]. Besides their basic functions as structural support materials in plants and as energy substances in living organisms, i.e., cellulose and starch, respectively, polysaccharides have a wide range of pharmacological effects, including anticoagulation, antibacterial, immunoregulatory, and antiviral effects [3]. A sulfated polysaccharide (SP), as a common, functional naturally derived polysaccharide, is a heterogeneous group of macromolecules with sulfate groups in its sugar residues that usually contains glyoxylates [4]. Due to the mature preparation method, SP is mainly extracted from aquatic plants such as *Arthrospira* [5].

*Arthrospira*, a tiny filamentous cyanobacterium, is one of the most studied cyanobacteria and is mostly grown commercially; it is also used as a food supplement [6]. Moreover, *Arthrospira* has been considered for many years as an important source of valuable eco-based extracts such as proteins and polysaccharides, which constitute 55–70% and 15–25% of the dry weight of *Arthrospira*, respectively [7,8]. Phycocyanins are the most common derivatives of *Arthrospira* and have applications in healthcare, cosmetics, and pharmaceuticals [9]. In contrast, polysaccharides have received relatively less attention. However, polysaccharides from *Arthrospira* sources, especially SP, have been reported to have potentially specific physico-chemical properties and a variety of important biological activities, including antiviral, immunomodulation, and antioxidant properties [10]. The activity of *Arthrospira* polysaccharides varies with the available forms and structure. Therefore, the development of a new *Arthrospira*-derived SP (AdSP) and related applications is still of great potential in biomedical applications.

The application of AdSP is usually as a drug, which is independent of the material system [11]. The difficulty of AdSP to associate with other polymers or material surfaces may be related to the abundant hydrophilic groups, obscuring the small number of functional groups that can form bonds with other materials. Meanwhile, published articles on the effects of AdSP on cell behavior are mainly focused on the inhibition of tumor cell proliferation, anti-angiogenesis, enhancement of hematopoietic stem cells, etc. [12,13,14]. Only one study focuses on the effects of AdSP on the behavior of neural stem cells [15]. Meanwhile, there are many recent studies on the preparation of composite materials, especially nanomaterials and hydrogels, with AdSP or modified AdSP [16]. Hydrogels with the addition of AdSP can be endowed with the characteristic properties of SP [17,18,19]. This also offers the possibility to utilize AdSP in cell culture and tissue engineering.

In recent years, some studies exist on the preparation of composite nanomaterials using AdSP [20], but they are mostly on nanoemulsions prepared by phase transformation [21,22] and rarely on aqueous synthesis systems or the role of AdSP as a modifier on nanoparticles [23,24]. Meanwhile, multifunctional AdSP has not been used as a surface coating material alone, probably due to its strong hydrophilicity and good water solubility. In the present study, we propose a new promising AdSP and explore its ability to promote the behavior of neural stem cells in three-dimensional hydrogels. The prospective formulation from the new AdSP, including the preparation of composite nanoparticles with other polysaccharides at room temperature with a potential drug-delivery function, and the coating materials for material surface properties were also evaluated.

## 2. Results and Discussion

### 2.1. Basic Properties of AdSP

**Figure 1:**
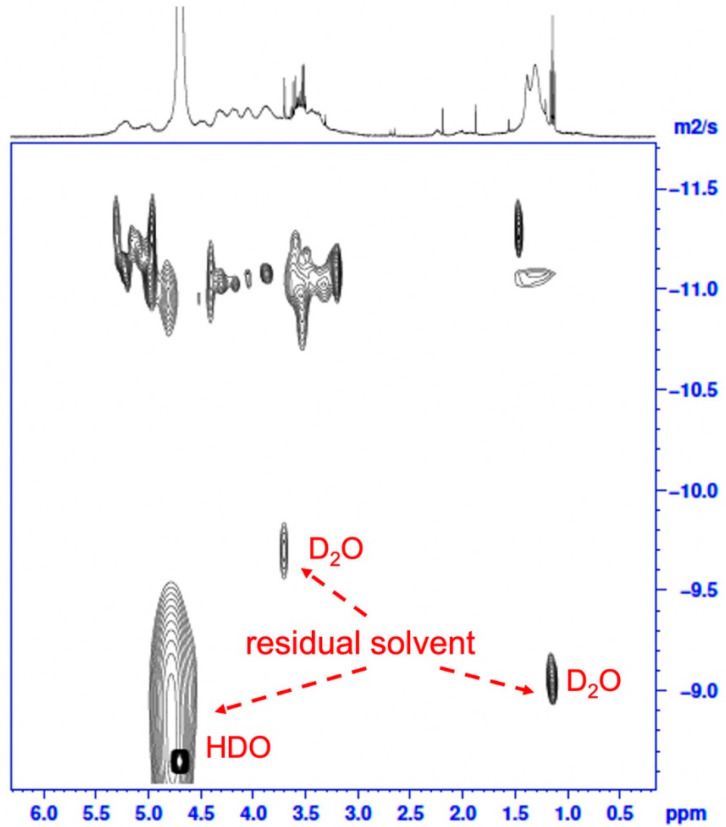
The two-dimensional DOSY spectrum of AdSP. The *X*-axis corresponds to the chemical shift in classic ^1^H NMR, and the *Y*-axis corresponds to the diffusion dimension.

The AdSP in the present study is a natural polysaccharide with a broad molecular mass distribution and a variable monosaccharide arrangement, which could be concluded from the previous studies discussing the diversity of *Arthrospira*-derived polysaccharides [25]. The molecular weight (M_w_) distribution of AdSP was confirmed through high-performance size-exclusion chromatography with refractive-index detection (HPSEC-RI), as shown in Figure S2 (Supplementary Materials). The AdSP presented a single-peak distribution with an average molecular weight of ~272 kDa, indicating the high homogeneity of the polysaccharide. Although there could be reasonable variations in the structure of AdSP, the purity of the polysaccharide should be clarified. As an attempt to decipher the structure of AdSP and confirm its purity, two-dimensional diffusion-ordered spectroscopy (DOSY), along with classic nuclear magnetic resonance spectroscopy (^1^H NMR), was employed, and the results are shown in Figure 1. From the classical ^1^H NMR (upper part of Figure 1), the arrangement of the monosaccharides of AdSP appeared to be promiscuous, so that the characteristic peaks overlapped considerably, which made it unlikely to justify a specific structure. However, the purity of the AdSP was verified by DOSY, excluding the effect of the residual solvent (D_2_O and HDO, as shown in Figure 1). The structural heterogeneity of AdSP may account for the irregular shape in the diffusion pattern of the DOSY result. These findings indicated that the AdSP developed in this work was a high-purity bio-sourced polymer.

An important feature of AdSP, i.e., the zeta potential, was investigated to provide a basis for further design of the materials. The zeta potential of AdSP was −42.87 ± 1.80 mV. Such a negative value indicates the good stability of AdSP in aqueous solution. The negative potential may be attributed to the rich hydroxy groups on the polysaccharide chain. In addition, the strong negatively charge of AdSP may allow it to readily interact with other molecules by electrostatic interaction. The electrostatic interaction can offer an effective way for AdSP to combine with other polymers to create composite materials.

The small-angle X-ray scattering (SAXS) technique was used to characterize the structural feature of AdSP. The scattering profile is shown in Figure S3 (Supplementary Materials). AdSP did not show a specific shape in the nanoscale. In addition, AdSP had various structures within two different scales (separated by 0.03 Å^−1^ in the curve). Due to the complexity of the monosaccharide composition and the uncertain spatial structure of AdSP, the characteristics of AdSP may deserve further investigation.

### 2.2. Bioactivity of AdSP-Loaded Hydrogels on Cell Behavior

**Figure 2:**
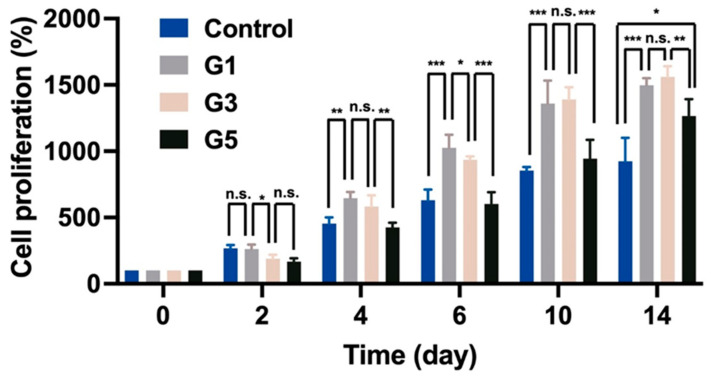
The proliferation of neural stem cells (NSCs) encapsulated in different hydrogels for a period of 14 days. * *p* < 0.05, ** *p* < 0.01, and *** *p* < 0.001 between the indicated groups.

Gelatin methacryloyl (GelMA) was successfully synthesized, and the degree of substitution (DS) of GelMA was verified through ^1^H NMR spectroscopy (Figure S4, Supplementary Materials). The DS was about 95%. GelMA hydrogel as a biocompatible and biodegradable hydrogel matrix has been widely used in biomedical fields [26]. Moreover, many previous works certified that GelMA hydrogel was a good supporting matrix for cell proliferation [27]. To examine the bioactivity of AdSP on cell behavior, three different AdSP-loaded GelMA hydrogels and a control hydrogel (GelMA hydrogel without AdSP) were produced for cell experiments, of which the component ratios and abbreviated names are listed in Table 1. The proliferation of neural stem cells (NSCs) encapsulated in hydrogel was analyzed by a Cell-counting Kit-8 (CCK-8) assay over a period of 14 days to assess the effects of AdSP on the behavior of NSCs. The results are shown in Figure 2. After 14 days, the G1 hydrogel group (with 1.0 mg/mL AdSP) and G3 hydrogel group (with 3.0 mg/mL AdSP) demonstrated a better proliferation rate of NSCs than the control hydrogel group (without AdSP) and the G5 hydrogel group (with 5.0 mg/mL AdSP). No significant difference existed between the G1 and G3 groups. All three AdSP-containing groups showed obvious promotion on the proliferation of NSCs compared to the control group. As the concentration of AdSP in the hydrogel increased, the proliferation rate of NSCs in the hydrogel did not show a completely positive correlation. When the concentration of AdSP in the hydrogel was greater than 3 mg/mL, the effect of AdSP in promoting the proliferation of NSCs in the hydrogel was reduced. Previously reported AdSP has not been investigated individually for its proliferative effects on NSCs, and only one study showed that *Arthrospira* exhibited some proliferative effects on human NSCs [15]. Our finding on AdSP is consistent with the latter observation on *Arthrospira*.

**Figure 3:**
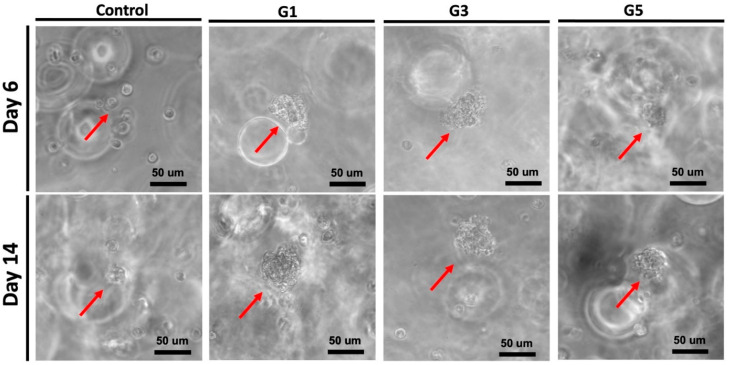
Potential clustering behavior of NSCs encapsulated in four different hydrogels on day 6 and day 14. Red arrows represent the neuro-clusters.

The potential clustering behavior of NSCs encapsulated in the hydrogels was observed by microscopy, as displayed in Figure 3. After 14 days, for all three AdSP-containing groups, cellular aggregation to variable extents was observed in the encapsulated NSCs. Eventually, cells were aggregated and formed multicellular spherical clusters. The average diameters of the NSC clusters in three AdSP-loaded hydrogels were ~58 μm, ~56μm, and ~39 μm for the G1 hydrogel, G3 hydrogel, and G5 hydrogel, respectively. The size of the single NSC used in this study was ~11 μm. However, the NSCs in the control group without AdSP did not show significant aggregation behavior. In the literature, AdSP has not been reported to present cell-aggregation-promoting activities. Meanwhile, such spherical clusters may have the potential to prolong stemness and maintain the multi-lineage of NSCs rather than separate cells [28,29]. Further gene expression assays were performed to verify the promotive effect of AdSP on cell behavior in hydrogel as well as its influence on gene expression.

**Figure 4:**
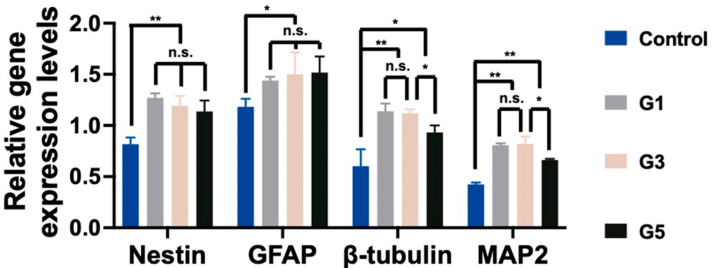
The stemness and differentiation of NSCs encapsulated in hydrogels. The gene expressions of neural-related genes, including nestin, GFAP, β-tubulin, and MAP2, were analyzed by RT-PCR after 14 days. The expression levels are represented by the relative ratios of gene expression normalized to that of GAPDH. * *p* < 0.05 and ** *p* < 0.01 between the indicated groups.

NSCs encapsulated in AdSP-loaded hydrogels and the control hydrogel (non-AdSP-containing) after a 14-day culture were investigated for their gene-expression profiles, to determine the differentiation status. The results are displayed in Figure 4. The expression levels of all genes, including the nestin (stemness marker), glial fibrillary acidic protein (GFAP, glial marker), β-tubulin (early neuronal marker), and microtubule-associated protein 2 (MAP2, mature neuronal marker) genes, showed significant differences between the AdSP-loaded hydrogels and the control group. No obvious difference existed among three AdSP-loaded hydrogels for the expression of nestin, indicating that spherical neuro-clusters probably maintain stemness longer than the the non-clustered NSCs in the control hydrogel. All AdSP-loaded hydrogels demonstrated no specific inclination for the differentiation direction of NSCs. The AdSP-loaded hydrogels tended to induce the differentiation of NSCs more toward neurons (β-tubulin and MAP2 genes) rather than glial cells (GFAP gene). This finding is consistent with the relevant literature, reporting that SP can promote neurosphere formation and may not influence the stemness and multi-lineage potential of neurospheres [30,31]. In addition, the high concentration of AdSP in the hydrogel (>5 mg/mL) decreased the expression level of neuron-related genes, though it remained higher than that of the control group. Therefore, an optimal concentration of AdSP for bioactivity on NSCs may exist, agreeing with the conclusion drawn by a cell-proliferation study. In the literature, a different type of AdSP was observed to possess possible neuro-protective effects in a Parkinsonian mouse model [32]. *Arthrospira* was also validated for its anti-inflammatory and inhibitory effects during the response of microglia to oxidative stress [15]. The new AdSP in this study, when in the appropriate range of concentration, can effectively promote the proliferation, spheroid formation, and differentiation of NSCs. This AdSP may, thus, be a promising candidate for the treatment of neurodegenerative diseases in the future.

### 2.3. Analyses of AdSP-Modified Substrates

A conventional overlay coating method was initially attempted to decorate AdSP on the substrate dish, which was conducted by overlay coating and washing three times, followed by drying [33]. The outcome showed that a significant difference between the dish treated by the overlay coating method and the untreated non-adhesive dish did not exist, indicating that AdSP was not readily attached to the substrate by the regular casting. The reason for this failure by the conventional coating may be attributed to the presence of numerous hydrophilic groups on the AdSP chain, which prevent it from binding to the substrate by simple contact. If AdSP successfully modifies the substrate surface, hydrophilicity and cytophilicity could be expected.

**Figure 5:**
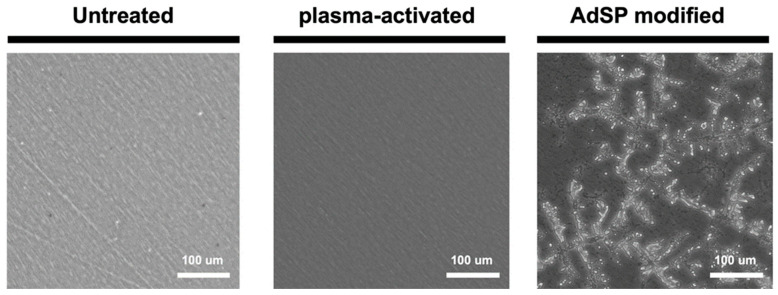
Surface images of the untreated non-adhesive dish, the plasma-activated dish, and the AdSP-modified dish observed by microscopy.

**Figure 6:**
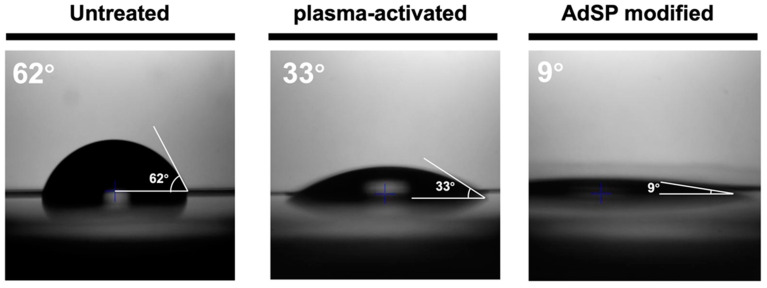
The average contact angle values for the untreated non-adhesive dish, the plasma-activated dish, and the AdSP-modified dish.

To graft AdSP onto the surface, a custom method was developed by using the air plasma treatment. The surface topography and hydrophilicity of the three groups, including a commercial non-adhesive polystyrene dish (untreated), plasma-activated dish, and AdSP-modified dish, were examined to determine if the surface modification of the substrates was successful. The surface of the untreated and plasma-treated dishes showed a similar morphology without a specific pattern. By contrast, the surface of the dried AdSP-modified dish exhibited a dendritic pattern in the substance attachment, as shown in Figure 5. After repeated moistening and drying, a similar dendritic pattern still remained on the surface of the ADSP-modified dish. In addition, the average values of the contact angles for the dishes shown in Figure 6 confirmed the difference among the substrates. The untreated dish and plasma-activated dish showed contact angles of ~62° and ~33°, respectively, indicating limited wettability. As for the AdSP-modified dish, a contact angle of less than 10° was detected, implying that the AdSP-modified surface possesses superhydrophilicity [34]. This result was also associated with the water solubility of AdSP itself. The changes in the surface morphology and hydrophilicity of the dish suggest the successful modification of the substrates by AdSP.

### 2.4. Cell Adhesion on AdSP-Modified Substrates

**Figure 7:**
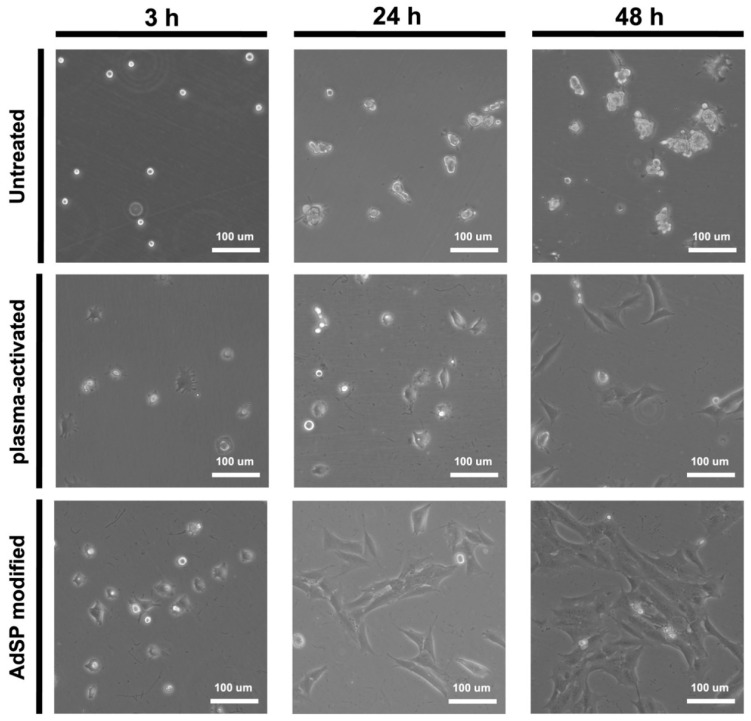
Cell adhesion behavior of NSCs within 48 h on the untreated non-adhesive dish, the plasma-activated dish, and the AdSP-modified dish.

NSCs were seeded on dishes in three different groups and then were observed by light microscopy at various pre-set time points within 48 h, as shown in Figure 7. About 80% of the NSCs seeded on the AdSP-modified dish exhibited adherent behavior, with a spreading cell morphology after a 3 h culture, and all NSCs showed complete attachment on the AdSP-modified dish after 24 h, along with slight proliferation. At 48 h, the NSCs on the AdSP-modified dish presented regular proliferation. On the contrary, NSCs were unable to adhere after 48 h of culture on the untreated dish and showed a wandering cell state; they were unstable and failed to grow in a continuous monolayer. Meanwhile, the NSCs growing on the plasma-activated dish attached to the surface slowly, with ~90% of the cells being adherent and morphologically stretched after 48 h but without obvious proliferation. The experimental results confirmed that AdSP coating could impart good cell adhesion to the surface of substrates. A different SP as the coating material has been used for antimicrobial and anti-fouling applications [35,36] but not for cell adhesion. Most commercially available tissue culture plates or dishes are made of polystyrene with surface modification such as hydrophilic polymer decoration or plasma treatment [37]. The finding in this study suggests AdSP as a promising coating option for substrates used in cell culture. Moreover, AdSP may have particular promotion effects in cell clustering and influence the differentiation behavior of some stem cells, based on the aforementioned results.

### 2.5. Potential of AdSP as Drug Carrier

**Figure 8:**
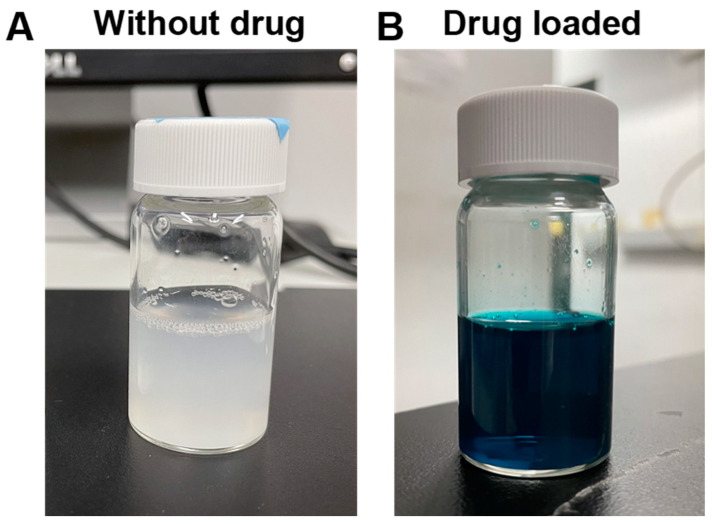
Photo images showing the suspension of composite nanoparticles based on AdSP and chitosan without model drug (**A**) and those loaded with drug (**B**).

Plant-resourced polysaccharide has been combined with a positively charged polymer to fabricate composite nanoparticles through the formation of a polyelectrolyte complex [38]. The potential of AdSP as a component to prepare composite nanoparticles was verified by a reaction with chitosan. A photo image of the suspension of AdSP/chitosan nanoparticles is shown in Figure 8A. The suspension was homogeneous with a milky-white semi-transparent appearance. The hydrodynamic diameter and polydispersity index (PDI) of the AdSP/chitosan nanoparticles were 239.67 ± 137.44 nm and 0.369, respectively. These preliminary features confirm the possibility of using AdSP as a component to prepare nanoparticles. The mechanism may be ascribed to electrostatic interaction and hydrophobic interaction [39]. The drug-loading capacity of AdSP/chitosan nanoparticles was subsequently verified using FG as a model drug. A photo image of the suspension of FG-loaded AdSP/chitosan nanoparticles is displayed in Figure 8B. The suspension was in the form of a blue-colored homogeneous semi-transparent dispersion. The hydrodynamic diameter and PDI of the FG-loaded AdSP/chitosan nanoparticles were altered to 306.17 ± 60.33 nm and 0.281, respectively. The drug-loading efficiency of the nanoparticle was estimated to be 18%, indicating the potential for drug delivery. The reason for the increment in the size of the drug-loaded particles was hypothesized to be the competition by the charge of drug in the electrostatic interaction between AdSP and chitosan that influences the regular self-assembly. Considering the properties of each composition in the formula, such as the pH sensitivity and antibacterial properties of chitosan [40] as well as the potential antiviral and immunomodulatory abilities of AdSP [10,11], the capabilities of AdSP/chitosan nanoparticles as a functional drug carrier may deserve further exploration, through the optimization of AdSP/chitosan nanoparticles for drug loading and release and the intensive study of their functions.

## 3. Materials and Methods

### 3.1. Materials

The purified sulfated polysaccharide (AdSP, polysaccharide content of ~99%, Mw = 272 kDa) extracted from self-grown *Arthrospira maxima* through the pressurized hot water extraction method was provided by Far East Bio-Tec. Co., Ltd. (Taipei, Taiwan). The molar ratio of each monosaccharide composition is listed in Table 2. Methyl acrylic anhydride (MAA), gelatin (type A, 300 Bloom, from porcine skin), deuterium oxide (D_2_O), and sodium bicarbonate were purchased from Sigma-Aldrich (St. Louis, MO, USA). High-glucose Dulbecco’s Modified Eagle’s Medium (HG-DMEM), Ham’s F-12, fetal bovine serum (FBS), and penicillin–streptomycin–amphotericin (PSA) were purchased from Gibco (Grand Island, NY, USA). Geneticin (G418) was purchased from Invitrogen (Waltham, MA, USA). 2,2-Azobis(2-methyl-*N*-(2-hydroxyethyl) propionamide) (VA-086) and FG were purchased from Wako Chemicals GmbH (Neuss, Germany). Chitosan (Mn~1.4 × 10^5^ Da) was obtained from Hopax lnc. (Taipei, Taiwan). CCK-8 (Sigma-Aldrich, St. Louis, MO, USA) and KAPA SYBR Green qPCR kit (Kapa Biosystems, Inc., Wilmington, MA, USA) were applied in the commercial form.

### 3.2. Characteristics of AdSP

AdSP, dissolved in D_2_O, was analyzed by the DOSY, along with classic n^1^H NMR (Bruker AVANCE III^TM^ HD 400 MHz NMR spectrometer, USA) operated at 300 K. The molecular weight analysis was determined by HPSEC-RI. Samples were separated by the TSKgel^®^ guard column PWH (7.5 mm × 7.5 cm, Tosoh Bioscience, Inc., Tokyo, Japan) coupled with the TSKgel^®^ G4000PW column (7.5 mm × 30 cm, Tosoh Bioscience, Inc., Tokyo, Japan) and TSKgel^®^ G3000PW column (7.5 mm × 30 cm, Tosoh Bioscience, Inc., Tokyo, Japan). Columns were eluted at a flow rate of 0.5 mL/min at 70 °C with 0.3 N NaNO_3_, containing 0.02% NaN_3_. The molecular weight was estimated with a calibration curve of the pullulan standard kit (P82, Lot:16021, Showa Denko America, Inc., New York, NY, USA). The ζ-potential of AdSP in aqueous solution was measured by a nanoparticle analyzer (Delsa Nano, Beckman Counter, Brea, CA, USA). The nanoscale structure of SP was investigated by SAXS, with the scattering vector (q)-range from 3 × 10^−3^ Å^−1^ to 2 × 10^−1^ Å^−1^ performed at the beamline station 23A of Taiwan Light Source (TLS 23A) at National Synchrotron Radiation Research Center (NSRRC), China.

### 3.3. Preparation of AdSP-Loaded Hydrogels

The synthetic procedure of GelMA followed the previous literature [41]. GelMA was prepared by dissolving gelatin (10 *w*/*v* %) in 100 mL of 0.25 M carbonate-bicarbonate buffer, and then 0.2 mL/g (MAA/gelatin) at 45 °C was slowly added under stirring for 90 min to achieve methacrylation. The resulting mixture was dialyzed through a dialysis membrane (MWCO = 12–14 kDa) in deionized water for 72 h and then freeze dried to obtain GelMA with a DS close to 95%. The obtained GelMA was dissolved in D_2_O and analyzed by NMR spectroscopy (^1^H NMR, Brucker AV III-500 MHz FT-NMR, USA) for the DS.

The composite hydrogel of GelMA and AdSP was then prepared. Both ingredients were completely dissolved in HG-DMEM and Ham’s F12 (1:1) with 1% sodium bicarbonate at 37 °C, and the VA-086 (photoinitiator) was then added into the prepolymer solution at 1.5 *w/v* % of total solid content under light-proof conditions. The weight percentage of GelMA in the prepolymer solution was 7.5 wt% with different concentrations of AdSP (0, 1, 3, and 5 mg/mL). The prepolymer was pipetted in the 24-well plate and exposed to UV light (22.4 mW/cm^2^, 360–480 nm) at the distance of 5 cm for 2 min to allow the formation of the photo-crosslinked network.

### 3.4. Cell Culture in AdSP-Loaded Hydrogels

NSCs (passage 7) used in this study were derived from adult mouse brain, as described in previous studies [42]. The cell medium for NSCs was a mixture of HG-DMEM and Ham’s F-12 (1:1) with 10% FBS, 400 μg/mL G418, and 1% PSA. NSCs were incubated in a humidified incubator at 37 °C and 5% CO_2_, and the medium was renewed every 2 days. For three-dimensional cell culture in hydrogels, NSCs with the density of 2 × 10^6^ cells/mL were suspended in the prepolymer solution before UV light exposure. The NSC-loaded hydrogels were placed into a 24-well plate for incubation. The proliferation of NSCs in hydrogels was assessed by the CCK-8 assay at pre-set time points for 14 days. Measurements with CCK-8 assay were performed using a SpectraMax M5 plate reader at the wavelength of 450 nm. The behavior of NSCs embedded in hydrogels was observed and captured by a light microscope coupled with a digital camera (Nikon, Eclipse 80i, Tokyo, Japan).

The expression levels of neural-related genes in NSCs embedded in hydrogels for 14 days were analyzed by real-time reverse transcriptase-polymerase chain reaction (RT-PCR) using the KAPA SYBR Green qPCR kit. The medium for NSCs in the evaluation of differentiation was G418-free culture medium. The results were detected and recorded using a Step One Plus Real-Time PCR instrument (Applied Biosystems, Waltham, MA, USA). The neurological markers used in this study included nestin, GFAP, β-tubulin, and MAP2. All primer sequences used in this research are shown in Table S1. The gene expression levels were normalized to glyceraldehyde 3-phosphate dehydrogenase (GAPDH) and then represented in relative proportions.

### 3.5. Preparation of AdSP-Modified Substrates

AdSP was coated on the material surface based on a self-developed method inspired from the previous literature [33]. The surface of non-adherent polystyrene dish (diameter/height: 35/10 mm, Greiner Bio-One, Frickenhausen, Germany) was used as the pristine substrate. The surface of the dish was first activated by air plasma scanning, and then the AdSP aqueous solution (1 mg/mL) was uniformly coated on the plasma-modified substrate for 1 min, followed by three washes with deionized water. The plasma was provided by a high-power open-air plasma system (Openair^®^) developed by Plasmatreat (Steinhagen, Germany). The plasma temperature at the nozzle outlet was ~26 °C, and the air pressure was 2.5 kg/cm^2^. The plasma power was set at 720 W. The substrate was placed at a distance of 200 mm from the nozzle, and the nozzle was scanned at a speed of 6 m/min. All samples were washed and dried at room temperature. The surface of the modified dish was observed by a light microscope coupled with a digital camera (Nikon, Eclipse 80i, Tokyo, Japan). Surface contact angles were measured at room temperature using an optical contact angle meter. To conduct the measurement, the dish was placed on a removable sample stage and leveled horizontally. A drop of deionized water was carefully placed on the surface, and the average of five measurements at different positions of the sample was recorded as the contact angle.

### 3.6. Cell Adhesion on AdSP-Modified Substrates

The source and culture conditions of NSCs were the same as described in Section 3.4. Before NSCs were seeded in a density of 5 × 10^4^ cells per dish, each dish was exposed to UV light for 24 h for sterilization. The cell morphology and adhesion on the dish surface were observed by light microscopy, where images were taken at pre-defined time points.

### 3.7. Evaluation of AdSP as a Component of Drug Carrier

The attempt to corroborate the use of AdSP as a nanocarrier was motivated by the previous literature [39]. Composite AdSP–chitosan nanoparticles were prepared by mixing the 1.0 mg/mL chitosan solution and 1.0 mg/mL AdSP solution at pH around 6.0 under the stirring speed of 600 rpm. The whole process to prepare composite nanoparticles was executed in room temperature. As for the drug-loading attempt, FG as a model drug was first added in the chitosan solution with a concentration of 500 ppm. Then, the AdSP solution was dropped in the chitosan/FG solution and mixed at 600 rpm. The target nanoparticles were obtained by centrifugation at the speed of 2500 rpm for 15 min. The PDI and hydrodynamic diameters of the nanoparticle and FG-loaded nanoparticle were analyzed by a nanoparticle analyzer (Delsa Nano, Beckman Counter). The loading efficiency was calculated through the formula (*W_i_* − *W_w_*)/*W_i_ ×* 100%, where *W_i_* is the initial weight of the drug, and *W_w_* is the weight of the unwrapped drug. The weight of the unwrapped drug was estimated in two steps. The absorbance in the supernatant after centrifugation was tested using a microplate reader (SpectraMax M5, Molecular Devices, San Jose, CA, USA) at the wavenumber of 552 nm for FG. Then, the concentration of FG was calculated through the absorbance and the calibration curve of FG (Figure S1, Supplementary Materials) to acquire the weight of the unwrapped drug.

### 3.8. Statistical Analysis

All quantitative results were obtained independently with at least three replicates to exclude unexpected cases (n ≥ 3). Computed data are expressed as mean ± standard deviation. Statistical differences were made between groups using the commercially distributed statistics software package GraphPad Prism 9 and a Student’s t-test, and data were deemed statistically meaningful if *p* < 0.05.

## 4. Conclusions

The new, high-purity sulfated polysaccharide extracted from *Arthrospira maxima* showed bioactivities. When added to the hydrogel, it effectively enhanced the proliferation and differentiation of embedded NSCs, along with prolonging the stemness. The formation of multicellular spherical cell clusters with diameters in a range of 40 to 60 μm was observed in the AdSP-containing hydrogel. An optimal concentration (less than 5 mg/mL) existed for the AdSP in the hydrogel to achieve the greatest ability in promoting cellular behavior. AdSP was also verified as serving as a coating material through the self-developed method for providing the AdSP-modified substrate with superhydrophilicity, i.e., a contact angle less than 10° and cell adhesiveness. In addition, AdSP was used as a component for preparing nanoparticles by simple mixing with chitosan at room temperature for drug-carrier applications. Such composite nanoparticles had an average diameter of about 240 nm, with a considerable drug-loading efficiency of ~18%. These findings suggest that the new AdSP proposed in this study may possess great potential in biomedical applications for cell culture, surface modification, and drug delivery.

## Figures and Tables

**Table 1:** Abbreviated names and compositions of the hydrogels.

Abbreviated Name	Concentration of GelMA (wt%)	Concentration of AdSP (mg/mL)
Control	7.5	0
G1	7.5	1.0
G3	7.5	3.0
G5	7.5	5.0

**Table 2:** Molar ratios (%) of monosaccharides in the composition of the *Arthrospira*-derived sulfated polysaccharide (AdSP) in this study.

Monosaccharide	Rhamnose	Glucose	Mannose	Fructose		Galactose
Molar ratio	91.3 ± 0.1	1.8 ± 0.2	1.2 ± 0.2	2.6 ± 0.1		1.7 ± 0.1
**Monosaccharide**	**Xylose**	**Arabinose**	**Glucuronic acid**		**Galacturonic acid**	
Molar ratio	Trace	Trace	0.2 ± 0.1		1.2 ± 0.5	

## Data Availability

Not applicable.

## Supplementary Materials

The following supporting information can be downloaded at: https://www.mdpi.com/article/10.3390/molecules28020732/s1. Table S1: The primer sequences for PCR used in this study; Figure S1: The calibration curve for the model drug fast green; Figure S2: The molecular weight distribution of AdSP, showing the high homogeneity of the purified sample; Figure S3: The SAXS profile of AdSP in the study; Figure S4: ^1^H NMR of GelMA prepared in the study.
