# Supplementary material for: Enhancement of Cell Behavior by the Polysaccharide Extract of Arthrospira and Potential Biomedical Applications
Source: Molecules. 2023 Jan 11;28(2):732. doi: 10.3390/molecules28020732 (PMC9863469; doi:10.3390/molecules28020732)
Supplement: Supplementary file 1 [file molecules-28-00732-s001.zip › molecules-2081196-supplementary.pdf]

Supporting information

Enhancement of Cell Behavior by the Polysaccharide Extract of *Arthrospira* and Potential Biomedical Applications

Junpeng Xu [1] and Shan-hui Hsu [1,2,*]

[1] Institute of Polymer Science and Engineering, National Taiwan University, No. 1, Sec. 4 Roosevelt Road, Taipei 10617, Taiwan
[2] Institute of Cellular and System Medicine, National Health Research Institutes, No. 35 Keyan Road, Miaoli 35053, Taiwan
* Correspondence: shhsu@ntu.edu.tw; Tel.: +886-2-3366-5313

Pages: 4

Tables: 1 (Table S1)

Figures: 4 (Figure S1-S4)

Table S1. The primer sequences used for RT-PCR analyses of mouse NSCs.

Gene	Primer	
	**Forward**	**Reverse**
mGADPH	GGCTACAGCAACAGGGTGGT	CGAGTTGGGATAGGGCCTCT
mNestin	ACTGTGGAATCACCAGGAGG	ATTCCACCTCTCCCAGAGAC
mGFAP	CTGAACCCTCTGAGCAAATG	GAATCAAACACAGAGCCTGC
mβ-Tubulin	CAGGGCCAAGACAAGCAGCA	GGAGCCCTAATGAGCTGGTGA
mMAP2	TTCTCCACTGTGGCTGTTTG	GAGCCTGTTTGTAGACTGGAAGA

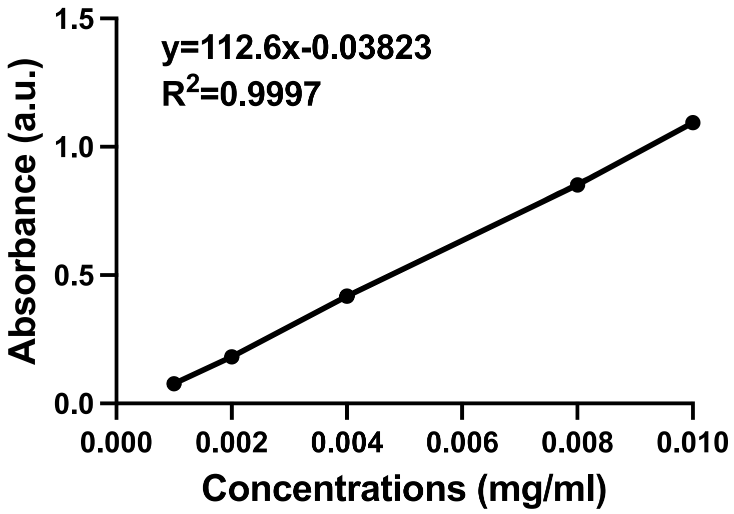

Figure S1. The calibration curve for the model drug fast green.

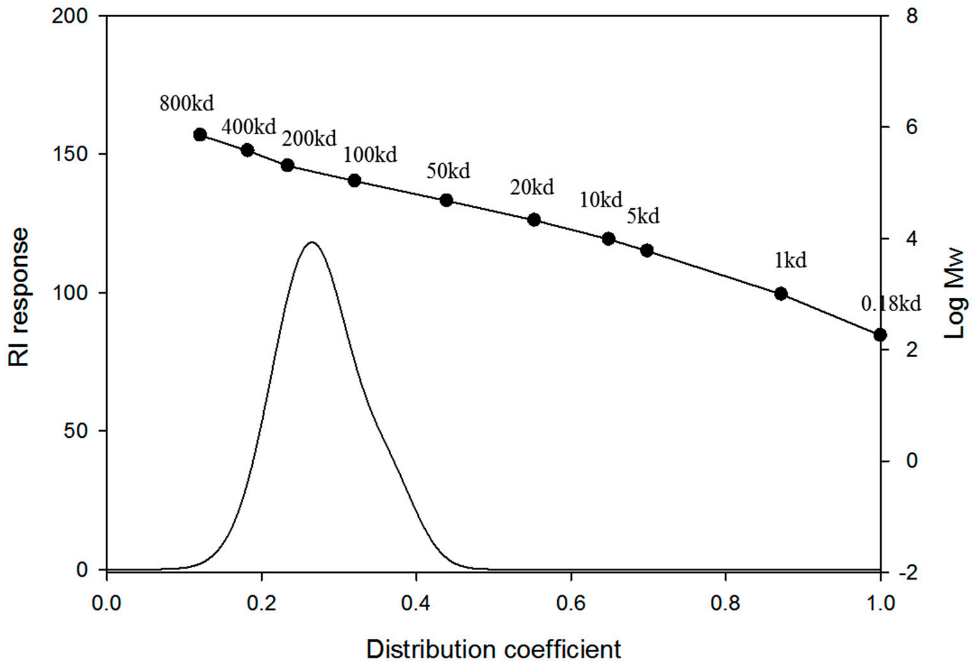

Figure S2. The molecular weight distribution of AdSP, showing the high homogeneity of the purified sample.

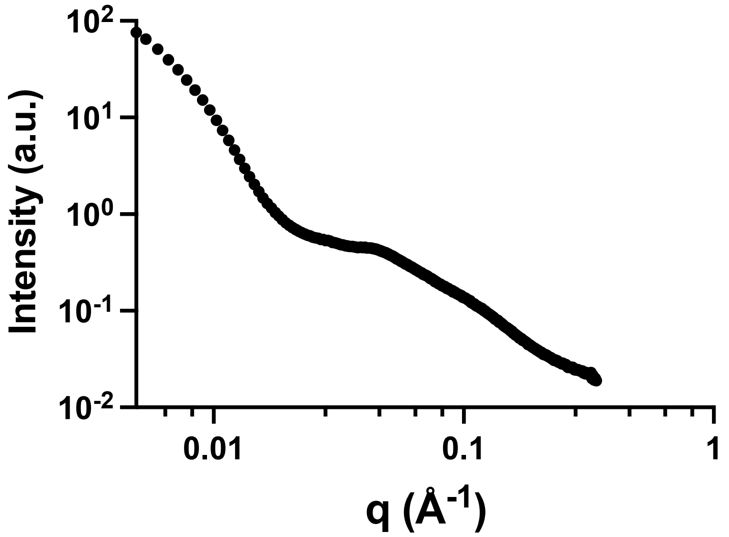

Figure S3. The SAXS profile of AdSP in the study.

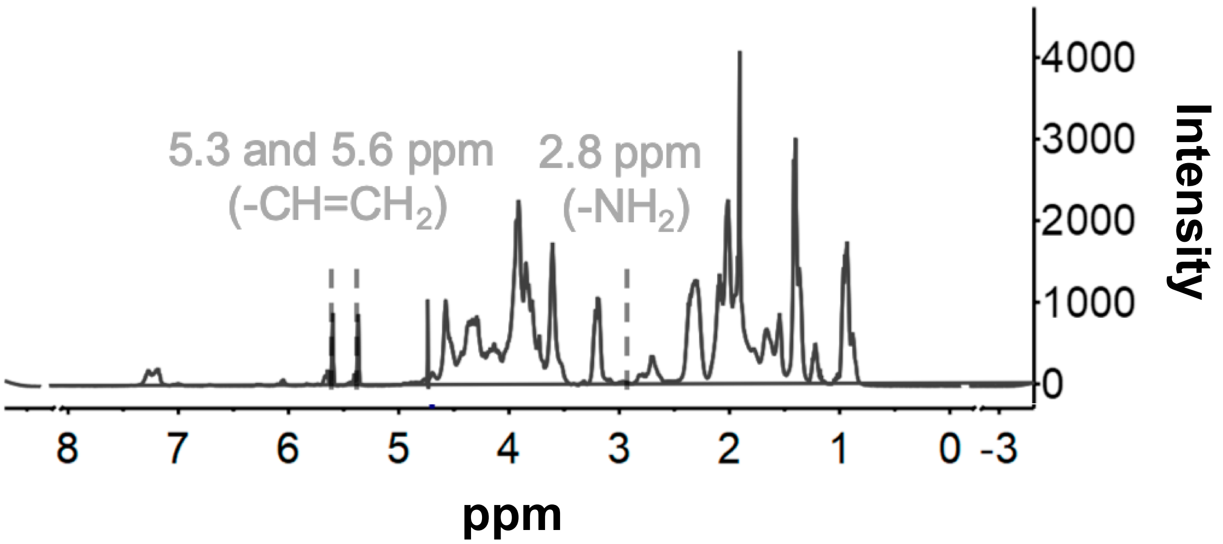

Figure S4. ^{1}H NMR of GelMA prepared in the study.
